# Systematic characterization of the expression, prognosis and immune characteristics of PLOD family genes in breast cancer

**DOI:** 10.18632/aging.206029

**Published:** 2024-07-26

**Authors:** Dan-Dan Wang, Lei Li, Yu-Qi Fu, Su-Jin Yang, Xiu Chen, Jun-Chen Hou, Qian Zhang, Xiao-Xue Tian, Jin-Hai Tang, Jian Zhang, He-Da Zhang

**Affiliations:** 1 Department of General Surgery, The First Affiliated Hospital of Nanjing Medical University, Nanjing 210029, China; 2 Department of Medical Oncology, The Affiliated Cancer Hospital of Nanjing Medical University and Jiangsu Cancer Hospital and Jiangsu Institute of Cancer Research, Nanjing 210009, China

**Keywords:** PLOD family genes, breast cancer, online databases, biomarker, tumor-infiltrating immune cells

## Abstract

Background: The expression patterns and prognostic value of Procollagen-lysine, 2-oxoglutarate 5-dioxygenase (PLOD) family genes in breast cancer remain to be elucidated.

Methods: The expression levels, prognostic value, and biological function of PLODs were determined using Oncomine, cBioPortal, GEPIA, Timer, UALCAN, PrognoScan, GeneMANIA, Metascape, and breast cancer tissue microarrays.

Results: The expressions of PLOD1 and PLOD3 were upregulated in breast cancer tissues, indicating worse clinical stages. High expression levels of PLOD family genes were associated with worse disease-free survival and distant metastasis-free survival, while high expression levels of PLOD1 and PLOD3 were related to worse overall survival in all breast cancer patients. The levels of PLOD family genes were all significantly higher in the age ≤51 y group, HR-negative patients, and triple negative breast cancer (TNBC) patients. They are associated with tumor-infiltrating immune cells (TIICs), including CD4+ T cells, CD8+ T cells, B cells, macrophages, neutrophils, and dendritic cells. According to co-expression gene analysis and functional enrichment, they are associated with protein hydroxylation, collagen biosynthesis and modifying enzymes, collagen metabolism, RNA splicing, extracellular matrix organization, VEGFA-VEGFR2 signaling pathway, and skeletal system development. Immunohistochemistry showed that the expressions of all PLOD family genes were significantly elevated in breast cancer tissues. PLOD1 expression was positively correlated with ER, TNBC status, and tumor grade. PLOD2 expression was positively connected with Ki-67 status. PLOD3 expression was positively related with age and tumor grade.

Conclusions: PLOD family genes are novel potential prognostic biomarkers for breast cancer, and targeting PLOD inhibitors might be an effective strategy for breast cancer therapy.

## INTRODUCTION

Breast cancer is the most common cancer and the leading cause of cancer-related death worldwide. Over 2,261,419 new breast cancer cases are diagnosed annually [1]. Although comprehensive development in surgery, chemotherapy, radiotherapy, endocrine therapy, and so on have improved the prognosis of breast cancer patients, there are no good biomarkers to predict the occurrence and progression of breast cancer. Therefore, it is urgent to develop good prognostic markers and effective drug targets for breast cancer treatment.

Procollagen-lysine 2-oxoglutarate 5-dioxygenases (PLODs), including PLOD1, PLOD2, and PLOD3, catalyze collagen cross-linking and deposition, depending on lysyl hydroxylation [2]. Dysregulation of PLODs is involved in the progression of multiple cancers, including proliferation, invasion, and metastasis [3, 4]. However, little is known about the expression patterns and functional roles of the PLOD family genes in breast cancer. Here for the first time, we aimed to discover the expressions and prognosis of PLOD family members in breast cancer tissues using various online databases and confirmed them through immunohistochemistry (IHC). This study might highlight novel biomarkers and effective target drugs for the treatment of breast cancer.

## MATERIALS AND METHODS

### Oncomine

Oncomine (http://www.oncomine.org/) is an integrated data-mining platform that collects cancer microarray data [5]. Oncomine was used to analyze the expressions of PLOD family genes (PLOD1, PLOD2, PLOD3) in different cancer tissues compared with normal tissues. The thresholds were as follows: *P*-value < 1E-4, fold change >2, and gene rank top 10%.

### cBio cancer genomics portal (cBioPortal)

cBioPortal (https://www.cbioportal.org/) for cancer genomics is a comprehensive and open accessible web resource that analyzes cancer genomic datasets, such as copy number variation, nonsynonymous mutations, mRNA and microRNA expressions, expressions and phosphorylation levels of proteins, DNA methylation, and clinical data [6].

### Gene expression profiling interactive analysis (GEPIA)

GEPIA (http://gepia.cancer-pku.cn/) is a newly developed interactive web server that estimates mRNA expression data from TCGA and GTEx [7]. Here, GEPIA was used to validate the differential expressions of PLOD family genes in breast cancer and healthy donor samples.

### Breast cancer gene-expression miner (bc-GenExMiner 4.6)

bc-GenGxMiner 4.6 (http://bcgenex.ico.unicancer.fr/) is a statistical mining tool based on published annotated breast cancer transcriptomic data including DNA microarrays and RAN-seq, and offers the possibility to explore gene expressions of genes of interest in breast cancer [8]. The expressions of PLOD family genes in different subtypes were analyzed using bc-GenGxMiner.

### UALCAN

UALCAN (http://ualcan.path.uab.edu) is a comprehensive and interactive web resource based on TCGA database [9]. We analyzed the relative transcriptional expressions of PLOD family genes in breast cancer and normal samples, and the expressions of PLOD family genes with clinical stages *P* < 0.01 was considered significant.

### PrognoScan

PrognoScan (http://www.prognoscan.org) is a new database for meta-analyzing the prognostic values of PLOD family genes in breast cancer. The recent availability of published cancer microarray datasets with clinical annotations facilitates the analysis of gene expressions for prognosis. *P* < 0.05 was regarded as meaningful.

### GeneMANIA

GeneMANIA (http://www.genemania.org) is a common website for constructing protein-protein interactions [10]. The online tool analyzes genes or gene lists through gene co-expression, gene co-location, gene enrichment analysis, physical interaction, and web prediction. We predicted the functions of PLOD family genes and visualized gene networks using GeneMANIA.

### Metascape

Metascape (http://metascape.org) is a well-maintained gene-list analysis tool for gene annotation and analysis using Gene Ontology (GO) and the Kyoto Encyclopedia of Genes and Genomes (KEGG) tools [11]. We used Metascape to conduct progress and pathway enrichment analyses of the PLOD family genes and neighboring genes.

### Tumor immune estimation resource (TIMER)

TIMER (https://cistrome.shinyapps.io/timer/) is a comprehensive analysis of tumor-infiltrating immune cells in various cancer and infers the abundance of tumor-infiltrating immune cells (TIICs) from TCGA [12]. We explored the expressions of the PLOD family genes of TIICs in breast cancer, including B cells, CD8+ T cells, CD4+ T cells, macrophages, neutrophils, and dendritic cells. In addition, we used immuneeconv to assess the reliable results of immune score evaluation. It is an R software package including MCP-counter.

### Tissue microarrays in IHC

IHC is the gold standard for detecting the expressions of *in situ* protein biomarkers in formalin-fixed, paraffin-embedded tumor tissues. First, tissue microarrays were baked at 60°C for 12 h and dewaxed in xylene, followed by gradient descent alcohol hydration. Sodium citrate was used for 6 minutes of antigen retrieval at 121°C. Endogenous enzyme blocking reagents and nonspecific blocking reagents (Kit-9710, Maixin, China) were used for incubation with primary antibodies at 4°C overnight in a wet box: PLOD1 (29480-1-AP, 1:100, Proteintech, China), PLOD2 (66342-1-Ig, 1:500, Proteintech, China) and PLOD3 (11027-1-AP, 1:500) (all Proteintech, China). On the second day, the assays were incubated with donkey anti-mouse/rabbit secondary antibodies and Streptomyces anti-biotin proteinperoxidase (Kit-9710, Maixin, China) for 10 min each. Finally, chromogenic detection was performed using a DBA detection kit (Kit-2031, Maixin, China) and nuclei were stained with hematoxylin. Additionally, the assays were dehydrated in a gradient concentration of ethanol and xylene and sealed with a neutral resin. IHC staining was scored by two independent pathologists, and the results were presented as levels (0, none; 1+, weak; 2+, moderate; 3+, strong) and the percentage of positively stained cells (0: <5%; 1:6–25%; 2:26–50%; 3:51–75%; and 4:75%). Finally, the score was expressed as (staining intensity × percentage of positively stained cells).

### Statistical analysis

Continuous variables were described as mean ± standard deviation (SD). Two and three groups were compared using independent sample *t*-test and one-way analysis of variance (ANOVA), respectively. Statistical and graphical analyses were done with SPSS 22 and GraphPad 8.0. *P* significance was set at *p* < 0.05.

### Data availability statement (DAS)

The authors confirm that the data supporting the findings of this study are available within its supplementary materials.

## RESULTS

### Expressions of PLOD1, PLOD2 and PLOD3 in different cancers

**Figure 1:**
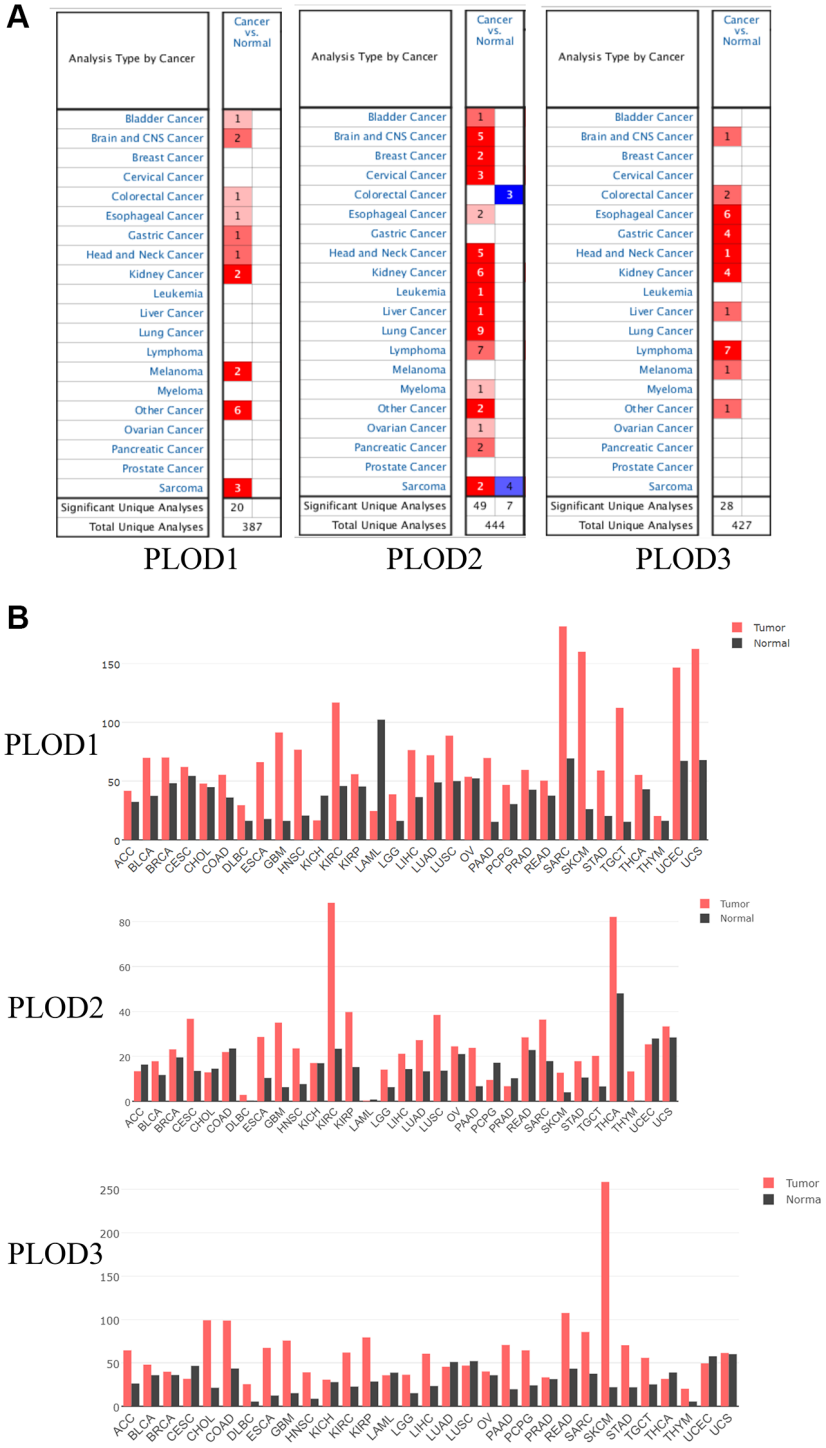
**The mRNA expression patterns of PLOD1, PLOD2 and PLOD3 in various cancers.** (**A**) The expression patterns were analyzed by Oncomine database; (**B**) The expression patterns were showed by GEPIA database.

We analyzed the mRNA expressions of PLOD1, PLOD2, and PLOD3 in various cancer and normal tissues using Oncomine database. The number of significant datasets for PLOD family genes was analyzed. Red and blue indicate overexpression and downregulation respectively. PLOD1, PLOD2, and PLOD3 levels were upregulated in most cancers (Figure 1A). Furthermore, the mRNA expressions of PLOD1, PLOD2, and PLOD3 were validated using GEPIA, which showed consistent results (Figure 1B).

### Expressions of PLOD1, PLOD2 and PLOD3 in breast cancer

**Figure 2:**
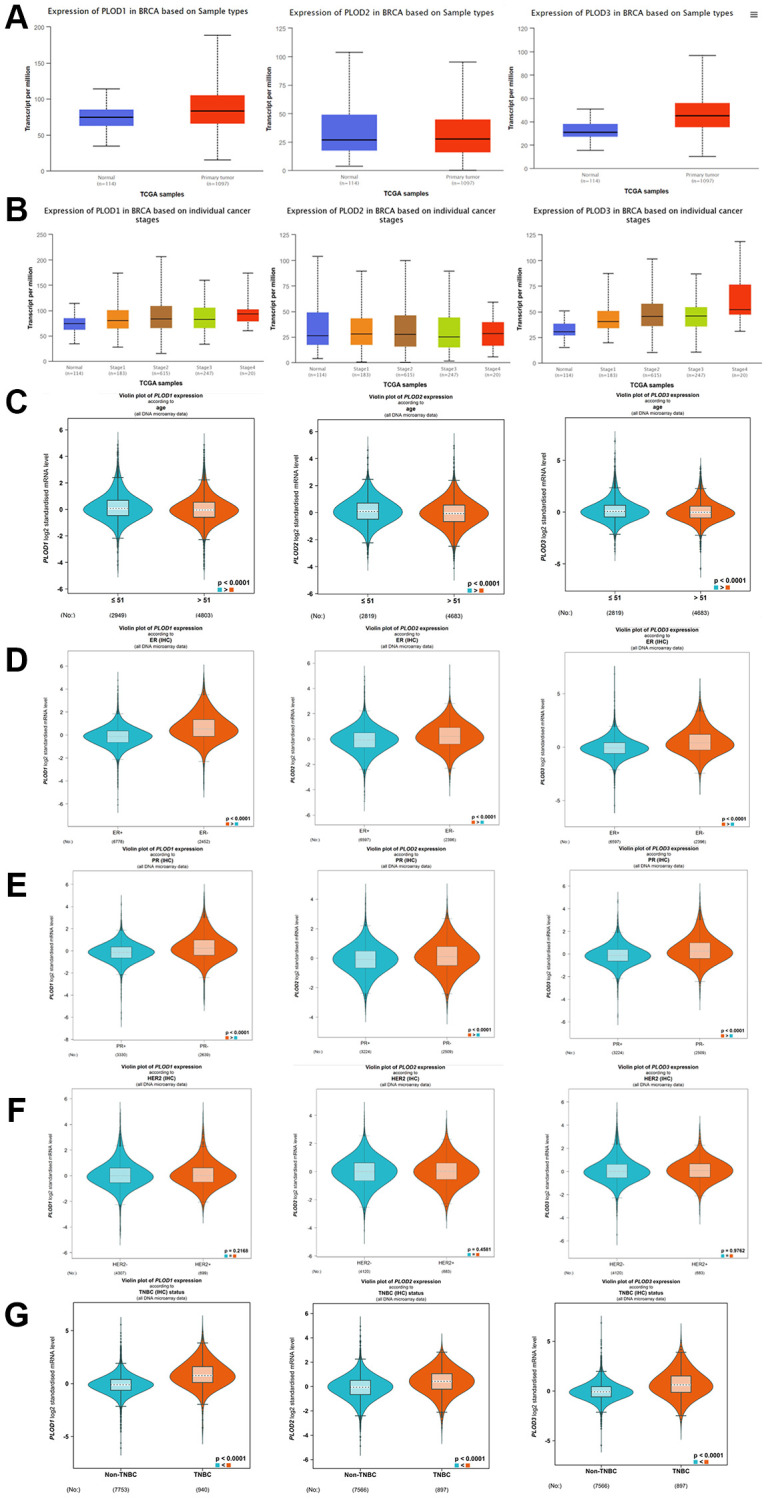
**The expression of PLOD family genes in breast cancer using UALCAN database.** (**A**) The expression of PLOD1, PLOD2 and PLOD3 in breast cancer tissues and normal tissues; (**B**) The expression of PLOD1, PLOD2 and PLOD3 in different clinical stages of breast cancer; bc-GeneExMiner 4.6 analyze the PLOD family genes in different clinical status. (**C**) Age; (**D**) ER; (**E**) PR; (**F**) Her-2; (**G**) TNBC.

Using UALCAN in TCGA, we discovered that PLOD1 and PLOD3 were significantly upregulated in breast cancer tissues compared to normal tissues (Figure 2A). In clinical stages, PLOD1 and PLOD3 were overexpressed in all stage subgroups compared with normal tissues (Figure 2B). However, PLOD2 expression was not significant in either of the above results (Figure 2A, 2B). In terms of age, the expressions of PLOD1, PLOD2, and PLOD3 were significantly higher in the ≤51 y group than in the >51 y group (Figure 2C). Regarding hormone receptor status, the expressions of PLOD1, PLOD2, and PLOD3 were upregulated in the estrogen receptor-negative (ER-) group versus the ER-positive (ER+) group and in the progesterone receptor-negative (PR-) group versus the PR-positive (PR+) group (Figure 2D, 2E). Additionally, no PLOD family gene was identified in the human epidermal growth factor receptor-2 (HER-2) status (Figure 2F). Besides, the levels of PLOD1, PLOD2 and PLOD3 were higher in triple negative breast cancer (TNBC) patients than non-TNBC patients (Figure 2G).

### Correlation of PLOD1, PLOD2 and PLOD3 expressions with prognosis in breast cancer

**Figure 3:**
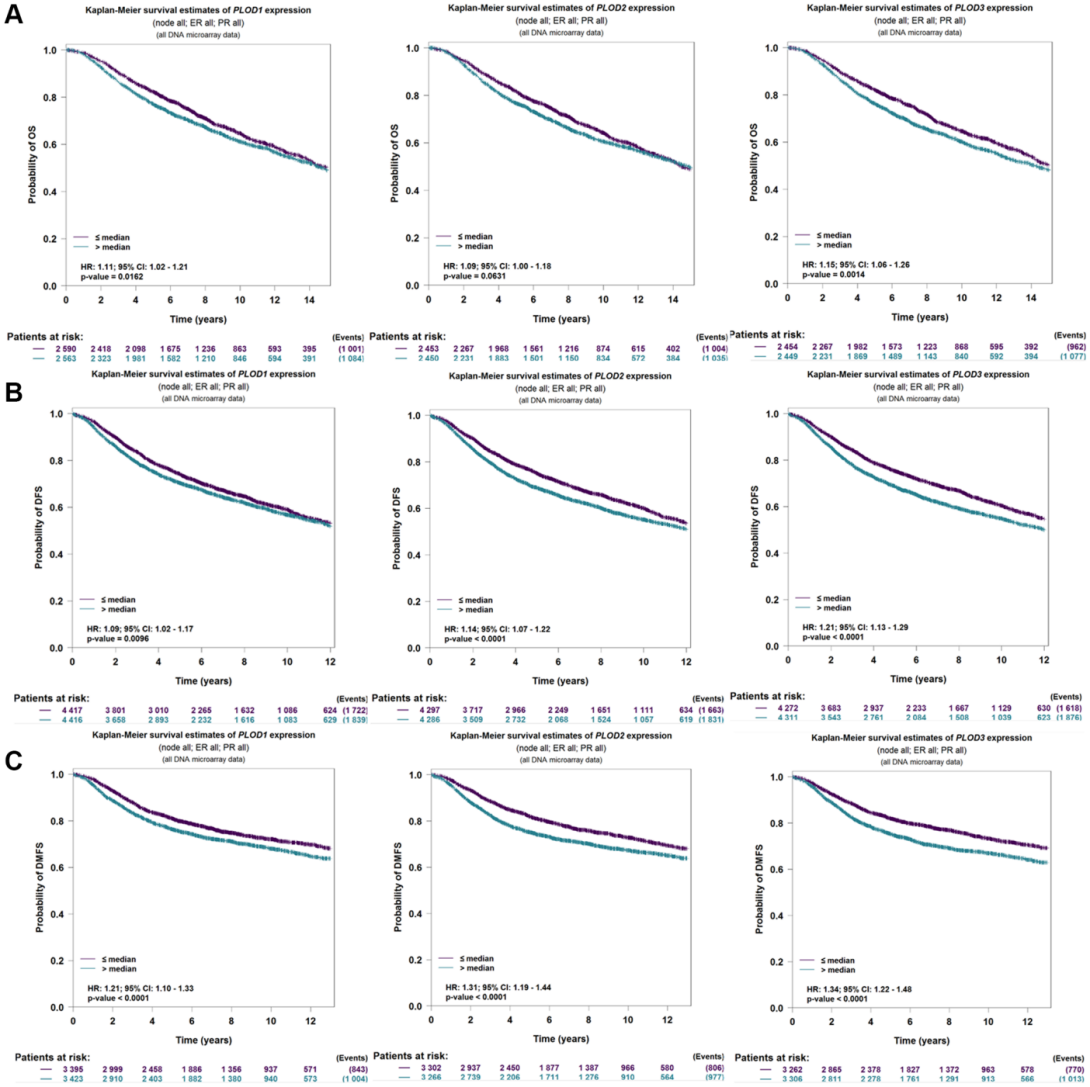
**The value of prognosis in PLOD family genes in breast cancer using bc-GeneExMiner 4.6 software.** (**A**) OS; (**B**) DFS; (**C**) DMSF.

We analyzed the prognostic value of PLOD family genes in bc-GeneExMiner 4.6. The Kaplan-Meier (KM) plotter showed that higher levels of PLOD1 and PLOD3 demonstrated worse overall survival (OS), whereas PLOD2 showed no difference in OS (Figure 3A). In addition, high expressions of PLOD1, PLOD2, and PLOD3 were associated with worse disease-free survival (DFS) and distant metastasis free survival (DMFS) (Figure 3B, 3C). Moreover, overexpression of PLOD1, PLOD2, and PLOD3 was associated with inferior DMFS, RFS, and OS (Table 1).

**Table 1:** PLOD family genes expression and survival data of breast cancer by PrognoScan database.

	**Dataset**	**End point**	**Probe ID**	***n***	***P*-value**	**HR**
PLOD1	GSE12276	RFS	200827_at	204	0.000416	1.47 (1.11–1.96)
	GSE11121	DMFS	200827_at	136	0.019318	0.45 (0.19–1.07)
	GSE1456-GPL96	OS	200827_at	159	0.049237	1.50 (0.71–3.18)
PLOD2	GSE12276	RFS	202619_at	204	0.046094	1.14 (0.94–1.38)
	GSE2034	OS	15487	155	0.18429	1.27 (1.08–1.49)
	GSE2034	DMFS	202620_s_at	286	0.001624	1.85 (1.39–2.47)
	GSE2990	DMFS	202619_s_at	125	0.042654	1.58 (0.95–2.63)
PLOD3	GSE9195	DMFS	202185_at	77	0.005538	4.48 (1.45–13.8)
	GSE9195	RFS	202185_at	77	0.00002	4.94 (1.89–12.96)
	GSE9893	OS	8888	155	0.00044	1.50 (1.15–1.96)
	GSE1456-GPL96	RFS	202185_at	159	0.046328	2.69 (1.35–5.38)

Abbreviations: RFS: Relapse free survival; DMSF: Distant metastasis free survival; OS: Overall survival.

### Expressions of PLOD1, PLOD2 and PLOD3 correlated with TIICs in breast cancer

**Figure 4:**
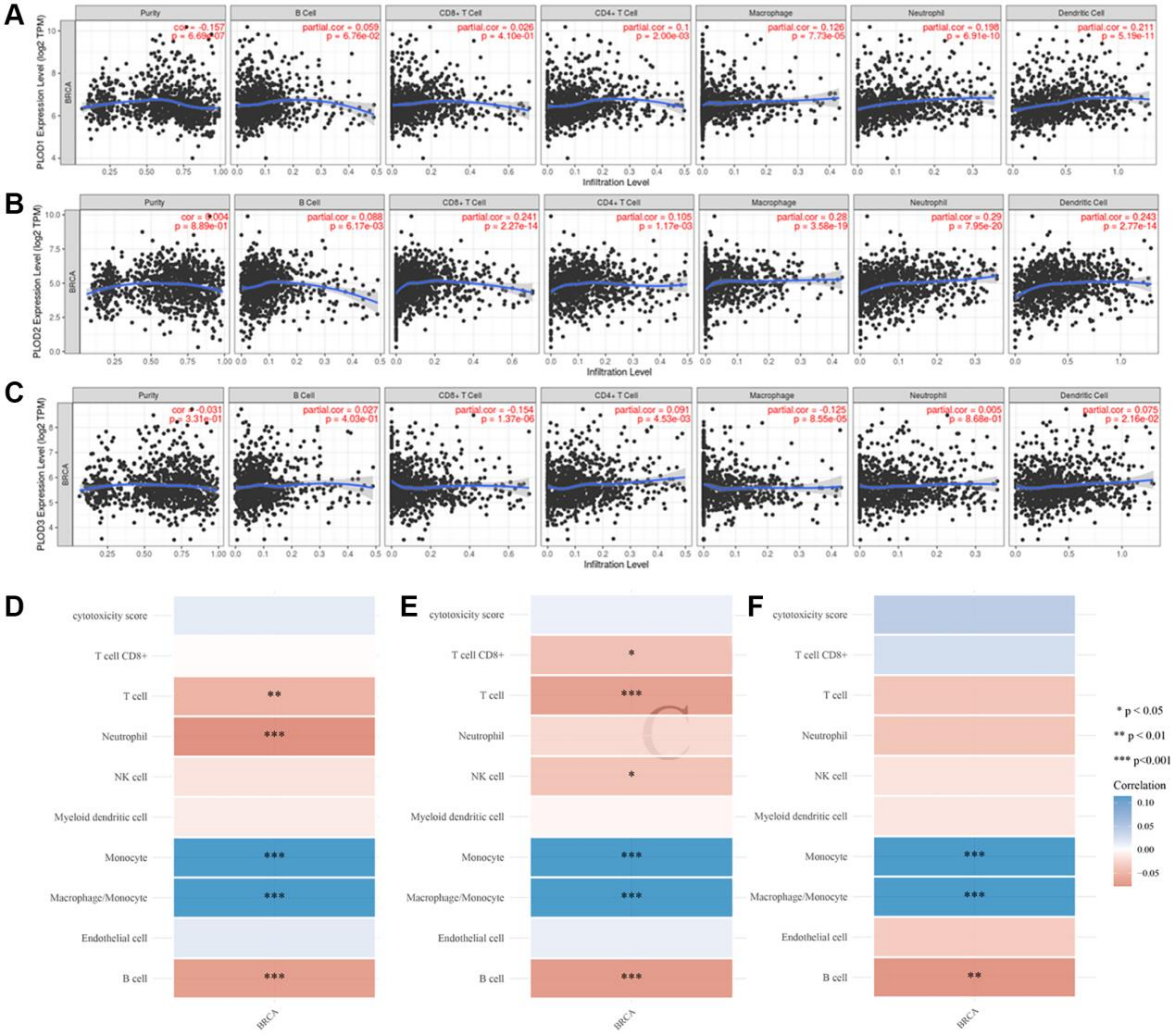
**Correlation of PLOD family genes with TIICs in breast cancer.** (**A**) The expression of PLOD1 in TIICs; (**B**) The expression of PLOD2 in TIICs; (**C**) The expression of PLOD3 in TIICs; (**D**) PLOD1 of immune score evaluation; (**E**) PLOD1 of immune score evaluation; (**F**) PLOD1 of immune score evaluation. Negative values indicate negative correlations and positive values indicate positive correlations, the deeper the color, the stronger the correlation. ^*^p < 0.05, ^**^p < 0.01 and ^***^p < 0.001, (^*^) stand for significance levels.

TIICs are an important complex in the tumor microenvironment of breast cancer and promote or suppress the development and growth of tumors. Therefore, we investigated PLOD family genes in TIICs of breast cancer. PLOD1 expression was positively correlated with the infiltrating levels of CD4+ T cells, macrophages, neutrophils, and dendritic cells (Figure 4A). PLOD2 expression was positively correlated with the infiltrating levels of B cells, CD8+ T cells, CD4+ T cells, macrophages, neutrophils, and dendritic cells (Figure 4B). PLOD3 expression was positively correlated with the infiltrating levels of CD8+ T cells, CD4+ T cells, macrophages, and dendritic cells (Figure 4C).

We further assess the reliable results of immune score evaluation through MCP-counter. PLOD1 was negatively correlated with monocyte (*P* < 0.001) and macrophage (*P* < 0.001), and positively correlated with T cell (*P* < 0.01), neutrophil (*P* < 0.001) and B cell (*P* < 0.001) (Figure 4D). PLOD2 was negatively correlated with monocyte (*P* < 0.001) and macrophage (*P* < 0.001) and positively correlated with CD8+ T cell (*P* < 0.05), T cell (*P* < 0.001), NK cell (*P* < 0.05) and B cell (*P* < 0.001) (Figure 4E). PLOD3 was negatively correlated with monocyte (*P* < 0.001), macrophage (*P* < 0.001), and was negatively correlated with B cell (*P* < 0.01) (Figure 4F).

### Co-expression, interaction analyses and functional enrichment analysis of PLOD family genes

**Figure 5:**
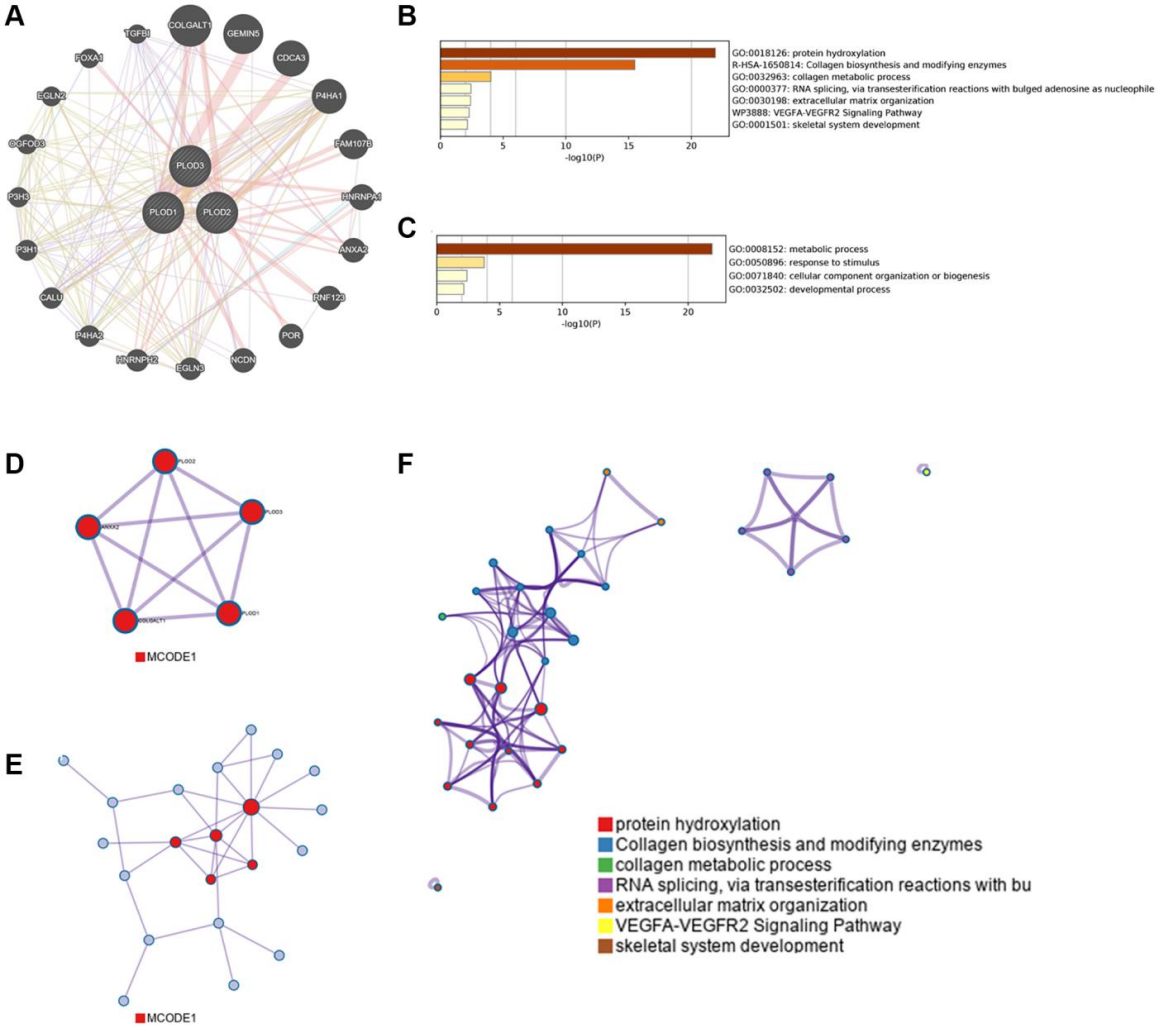
**Protein-protein interaction network in POLDs in GeneMANIA.** (**A**) Co-expression of PLOD family genes; (**B**) Heatmap of GO enriched terms and biological processes colored by the *p*-value; (**C**) Networks of GO-enriched terms and biological processes using the *p*-value; (**D**, **E**) Protein-protein interaction network and significant MCODE components from protein-protein network. (**F**) Independent functional enrichment analysis of MCODE components.

We analyzed the relationship in the co-expression of PLOD1, PLOD2, and PLOD3 at the gene level using GeneMANIA (Figure 5A). PLOD family genes and their neighboring top 20 genes were chosen and analyzed using GO and KEGG in Metascape. The co-expression was associated with protein hydroxylation, collagen biosynthesis and modifying enzymes, collagen metabolic processes, RNA splicing, extracellular matrix organization, the VEGFA-VEGFR2 signaling pathway, and skeletal system development (Figure 5B). It was also related to biological processes, such as metabolic processes, responses to stimuli, cellular component organization, and biogenesis and development processes (Figure 5C). To better understand the relationship among PLOD family members, we constructed a protein-protein interaction network and MCODE components using Metascape (Figure 5D–5F). Results showed the biological processes were mainly related to lysine degradation, collagen biosynthesis, and enzyme modification.

### Expressions of PLOD family genes in breast cancer tissues by IHC

**Figure 6:**
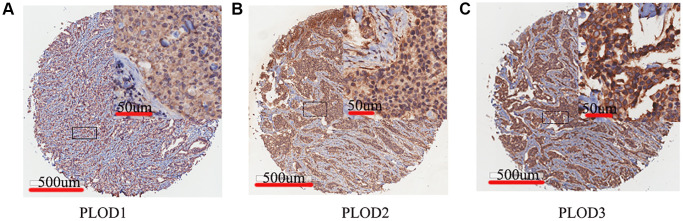
**Representative IHC staining of PLOD family genes in breast cancer.** Brown staining in cytomembrane and plasm indicates positive staining. (**A**) PLOD1; (**B**) PLOD2; (**C**) PLOD3.

To further validate the role of PLOD family genes in breast cancer progression, we examined their levels in breast cancer and non-tumor breast tissues using IHC. Tissue microarrays including 85 tumor and 85 para-tumor tissue samples were used here. PLOD1 and PLOD2 and PLOD3 were mainly expressed in the cytomembrane and plasma (Figure 6A–6C). IHC staining to show that the levels of all PLOD family proteins were significantly higher in breast cancer tissues than para-tumor tissues (Table 2, *P* < 0.001). In breast cancer tissues, the total immunostaining score of PLOD1 expression was significantly associated with the ER status (*P* = 0.04384), the TNBC status (*P* = 0.01592) and tumor grade (*P* = 0.001192). PLOD2 expression was positively correlated with the Ki-67 status (*P* = 0.01167). PLOD3 expression was positively correlated with age (*P* = 0.006977) and tumor grade (*P* = 0.0004829) (Table 3).

**Table 2:** The expression of PLOD family genes in breast cancer.

	**Breast cancer**	**Para-tumor**	***P*-value**
PLOD1	77	60	**<0.0001**
PLOD2	72	37	**<0.0001**
PLOD3	82	66	**<0.0001**

**Table 3:** Clinicopathologic characteristics of PLOD family genes expression in breast cancer tissues.

**Parameter**	**PLOD1**		***P*-value**	**PLOD2**		***P*-value**	**PLOD3**		***P*-value**
	**High**	**Low**		**High**	**Low**		**High**	**Low**	
**Age**									
≤51	17 (39.53%)	7 (46.67%)	0.6292	14 (46.67%)	4 (50%)	0.8668	14 (28.57%)	9 (69.23%)	**0.006977**
>51	26 (60.47%)	8 (53.33%)	16 (53.33%)	4 (50%)	35 (71.43%)	4 (30.77%)
**ER status**									
Positive	40 (93.02%)	11 (73.33%)	**0.04384**	26 (86.67%)	7 (87.5%)	0.9506	39 (79.59%)	12 (92.31%)	0.286
Negative	3 (6.98%)	4 (26.67%)	4 (13.33%)	1 (12.5%)	10 (20.41%)	1 (7.69%)
**PR status**									
Positive	37 (86.05%)	11 (73.33%)	0.2617	24 (80%)	7 (87.5%)	0.6268	13 (26.53%)	1 (7.69%)	0.1487
Negative	6 (13.95%)	4 (26.67%)	6 (20%)	1 (12.5%)	36 (73.47%)	12 (92.31%)
**HER2**									
Positive	9 (20.93%)	13 (86.67%)	0.5181	6 (20%)	0 (0%)	0.1681	11 (22.45%)	2 (15.38%)	0.578
Negative	34 (79.07%)	13 (86.67%)	24 (80%)	8 (100%)	38 (77.55%)	11 (84.62%)
**Ki67**									
≤14	2 (4.65%)	2 (14.29%)	0.2203	2 (6.67%)	3 (42.86%)	**0.01167**	3 (6.12%)	2 (16.67%)	0.2327
>14	41 (95.35%)	12 (85.71%)	28 (93.33%)	4 (57.14%)	46 (93.88%)	10 (83.33%)
**TNBC**									
TNBC	2 (4.65%)	4 (26.67%)	**0.01592**	4 (13.33%)	1 (12.5%)	0.9506	8 (16.33%)	1 (7.69%)	0.4321
Non-TNBC	41 (95.35%)	11 (73.33%)	26 (86.67%)	7 (87.5%)	41 (83.67%)	12 (92.31%)
**TNM**									
I	18 (43.9%)	11 (64.71%)	**0.001192**	13 (43.33%)	5 (62.5%)	0.09947	17 (36.96%)	8 (50%)	**0.0004829**
II	4 (9.76%)	6 (35.29%)	2 (6.67%)	2 (25%)	4 (8.7%)	7 (43.75%)
III	19 (46.34%)	0 (0%)	15 (50%)	1 (12.5%)	25 (54.35%)	1 (6.25%)

## DISCUSSION

The PLOD family genes encode the lysyl hydroxylase protein, which is involved in collagen biosynthesis. Dysregulation of PLOD family genes has been linked to various cancers, such as breast cancer, bladder cancer, esophageal squamous cell carcinoma, and hepatocellular carcinoma [13–16]. The roles of the PLOD gene family in several cancers have been well documented, but bioinformatic analysis has not been performed in breast cancer. Here, we determined the expression levels, prognostic value, TIICs, co-expression, and functional pathway of all PLOD family genes, which we propose as potential prognostic biomarkers for breast cancer.

We found PLOD1 and PLOD3 were highly expressed in breast cancer tissues and in all stage subgroups. All the PLOD family genes were upregulated in the HR-negative and TNBC groups. TNBC does not respond to hormonal receptors orHER-2 and has poorer prognosis than other breast cancer types. We showed that all PLOD family genes were highly expressed in TNBC, which provides evidence that they might be biomarkers and targets for TNBC. Wang et al. confirmed that PLOD3 was upregulated in gastric cancer and was associated with a larger tumor size, which could inhibit cell proliferation [17]. We obtained the same result, which reminds us to further confirm the function of PLOD family genes in breast cancer, particularly in a wide array of tumors. Multiple studied identified high expressed PLOD family genes as tumor promotors influenced cancer phenotype, however, the efficacy of targeting PLOD genes as a therapeutic strategy in breast cancer patients with high PLOD expression levels are needed more investigations.

To investigate the correlation between PLOD family genes and prognostic values in breast cancer, we further proved that high expressions of PLOD1 and PLOD3 were markedly related to worse OS, and all PLOD family genes represented worse DFS and DMFS. Recent studies found that high levels of PLOD1 and PLOD3 were related to short OS in gastric cancer, and high levels of PLOD1 and PLOD2 were related to poor OS in bladder cancer [18, 19]. In summary, these findings suggest that PLOD family genes might be tumor-promoting oncogenes, and might serve as diagnostic and prognostic biomarkers in breast cancer.

Co-expression and pathway analyses reveal that PLOD family genes mainly participate in lysine degradation, collagen biosynthesis, and enzyme modification. PLOD1 is upregulated in gastric cancer tissues and promotes tumorigenesis by activating the SOX9/PI3K/Akt/mTOR pathway [20]. Overexpression of the cell adhesion molecule L1 induces higher expression of ezrin-dependent PLOD2 by reducing SMAD2/3 in colon cancer, which stimulates cell proliferation, tumorigenesis, and liver metastasis [21]. PLOD3 decreases trastuzumab sensitivity by repressing FOXO3, resulting in the upregulation of Survivin protein in gastric cancer [22]. However, the functional roles of PLOD family genes in the cell proliferation, apoptosis, invasion, metastasis and tumorigenesis of breast cancer are not well understood. Therefore, the above findings lead us to extend research on the biological functions of PLOD family genes in breast cancer.

An increasing number of studies show that TIICs are significant predictors of immunotherapy and its clinical outcomes. Our study demonstrates that different PLOD family genes are associated with different TIICs, such as CD4+ T cells, macrophages, neutrophils, dendritic cells, B cells, and CD8+ T cells. As reported, PLOD2 plays an important role in TIICs of osteosarcoma, such as macrophages, CD8+ T cells, DC, B cells, and Th1cells [23]. Chen et al. confirm that PLOD family genes activate TIICs and correlate with the immune response in bladder cancer [19]. Furthermore, TIICs in breast cancer can be used to guide clinical immunotherapy. Taken together, we hypothesize that the PLOD gene family is markedly related to the tumor immune microenvironment in breast cancer and acts as a vital modulator in tumorigenesis. These observations suggest that PLOD family genes could be new potential targets for immunotherapy in breast cancer.

We specifically point out that the associations between the expressions of PLOD family genes in our tissue microassay and clinical characteristics are not exactly the same as those in online databases. The reasons may be that we had a different stuff or a smaller sample size. Such differences remind us that any public database needs validation by our objective data and calls for further molecular mechanisms to confirm its functions.

Objectively, there are also some limitations in our study. Although we described the expression of by all PLOD family genes in tissue microarrays, the prognostic prediction of that was accorded by TCGA database. Our study only showed the relationships between mRNA expression levels of PLOD family genes and breast cancer, indeed, the protein expression of these is also important. Moreover, we confirmed the co-expression on PLOD family genes in breast cancer tissues, however, the exact molecular mechanisms had not been validated in cellular or animal level and whether the pathway could be used as target therapy.

In summary, overexpressed PLOD family genes are associated with poor prognosis in breast cancer patients and the tumor immune microenvironment, and thus are superior prognostic indicators for breast cancer patients.
